# Could a Factor That Does Not Affect Egg Recognition Influence the Decision of Rejection?

**DOI:** 10.1371/journal.pone.0135624

**Published:** 2015-08-21

**Authors:** Francisco Ruiz-Raya, Manuel Soler, Lucía Ll. Sánchez-Pérez, Juan Diego Ibáñez-Álamo

**Affiliations:** 1 Dept. Zoology, Facultad de Ciencias, Universidad de Granada, Granada, Spain; 2 Grupo Coevolución, Unidad Asociada al CSIC, Universidad de Granada, Granada, Spain; 3 Animal Ecology Group, Centre for Ecological and Evolutionary Studies, University of Groningen, Groningen, The Netherlands; 4 Dept. Wetland Ecology, Estación Biológica de Doñana, CSIC, Sevilla, Spain; Institut Pluridisciplinaire Hubert Curien, FRANCE

## Abstract

Rejection of the parasitic egg is the most important defence of hosts against brood parasites. However, this response is variable among and within species, and egg discrimination is not always followed by egg rejection. Low risk of parasitism and high risk of rejection costs may lead to the acceptance of the parasitic egg even if it has been previously recognized. The main aim of this paper is to answer a relevant question: can a single egg trait provoke the acceptance of an experimental egg previously recognized as foreign? Increased egg mass should hamper the ejection of an egg that has been discriminated because ejection of a heavy egg may imply higher rejection costs for hosts. We have tested this prediction by experimentally parasitizing natural nests of Common Blackbirds (*Turdus merula*) with non-mimetic model eggs of different mass (heavy, normal-weight, and light) while controlling for potential confounding factors such as egg size and colour. Our results showed that blackbirds more frequently accepted heavy eggs, even when previously recognized. This differential acceptance may be related to insufficient motivation to assume the higher costs that the ejection of a heavy egg could impose.

## Introduction

Avian brood parasites lay their eggs in the nest of other species (hosts), thereby imposing high fitness costs on parasitized individuals because the newly hatched parasitic chick is usually better at competing for food or can directly eliminate competition by ejecting or killing the host offspring [1, 2]. Accordingly, there is strong selective pressure on hosts to evolve defences in response to parasitism, such as nest defence or egg discrimination and rejection [3, 4]. In response to this, brood parasites have evolved counter-defences to defeat host strategies, such as secretive laying or egg mimicry, resulting in a coevolutionary arms race between brood parasites and hosts [5], which makes the brood parasite-host system one of the clearest examples of coevolution found in nature [1, 2].

Rejection of parasitic eggs is the most widespread defence among hosts [1, 2, 6] and consequently it has been the focus of many studies in the field of avian host-brood parasite interactions. Egg-recognition experiments have led to a breakthrough in our understanding of the mechanisms involved in egg recognition and rejection and their evolutionary implications, e.g. [3, 4, 7–13]. Ejection of the parasitic egg and desertion of the parasitized nest are the two main mechanisms of egg rejection used by hosts. Egg ejection implies removing the parasitic egg from the nest, either by directly grasping the egg with the bill (grasp ejection) or, alternatively, by making a hole in the egg’s shell, gripping it and flying away with the egg (puncture ejection) [2].

Most previous studies in brood parasitism have focused in the action component of the rejection process and most of them do not distinguish between recognition and rejection components. However, the rejection process is a complex behaviour in which the previous discrimination of the parasitic egg by hosts is the first necessary step for the process to result in successful ejection [13]. Egg recognition is closely linked to the evolutionary background of hosts and their adaptations to brood parasitism [14], but hosts also need to have the genetic basis and the cognitive abilities necessary to be able to “decide” whether to reject the parasitic egg or not [15]. In terms of colour and design, it is well known that hosts are more likely to reject non-mimetic eggs than mimetic ones [3, 7, 16–18], which selects for egg mimicry [3] provoking many cuckoo species to lay eggs with such successful mimicry that the human eye is unable to distinguish them from the host eggs [19, 20].

Although the rejection of foreign eggs is the most widespread defence among actual and potential hosts of the common cuckoo (*Cuculus canorus*), there is a considerable variation in the response to parasitic eggs both among and within species [21–25]. Intra-specific variation in this respect indicates that genetic support and cognitive abilities involved in the recognition process are not the only factors responsible for the rejection of parasite eggs. Indeed, several studies have shown that egg discrimination is not necessarily followed by egg rejection [13, 24, 26–29], signifying that rejection is a plastic response, as previously suggested by theoretical models as well as empirical data [13, 25, 30–32].

Given that during rejection events hosts may accidentally break their own eggs (ejection costs) or eject a host egg by mistake (recognition costs) [2], the decision of whether to reject or not depends on a trade-off between costs and motivation, so that greater incentive is needed to assume higher costs [13]. Previous studies have suggested that the acceptance of recognized eggs is due to the impossibility of puncturing the parasitic egg by small-sized hosts, indicating that some egg traits (i.e. eggshell strength) may critically influence the acceptance of a previously recognized egg [29]. However, very little is known about the role of other egg traits that could affect the egg-rejection decision. In this regard, egg mass is one of these poorly studied trait. Egg mass and egg size are highly correlated (normally r^2^ > 0.8; [33]), so the importance of egg mass by itself in the ejection of the parasitic egg is not usually considered. It is possible that an egg with greater mass will be more difficult for grasp ejectors to take away from the nest due to greater difficulty in egg handling. While egg mimicry and egg size relative to the host- egg size can affect egg recognition, it is likely that egg mass alone cannot. The high degree of correlation between size and mass (see above) leads recognition to rely on egg size instead of the mass. Furthermore, open-nesting hosts (such as blackbirds) normally rely on visual cues to discriminate against parasitic eggs instead of tactile cues, which are preferably used by some dome-nesting hosts where the lower light availability make it harder to visually identify the alien egg [14].

The main aim of this study was to experimentally determine whether a single egg trait (i.e. egg mass), which does not affect egg recognition, can influence egg rejection decisions in the Common Blackbird (*Turdus merula*), a grasp-ejector species. For this purpose, we designed an experimental study in which blackbird nests were parasitized with non-mimetic blackbird eggs with exactly the same colour and size, but different weight, thus ensuring that introduced eggs were easily recognized (i.e. non-mimetic eggs). We predict that (1) non-mimetic model eggs will be equally recognized by blackbirds regardless of egg mass; (2) differences in egg mass will influence the acceptance of model eggs previously recognized: blackbirds will find it harder to eject heavy eggs and, as a result, they will be more likely to be accepted than lighter eggs; and (3) the ejection of a heavy egg will imply ejection costs due to greater handling difficulties.

## Materials and Methods

### Study site and species

We conducted our study in the Valley of Lecrín (southern Spain, 36º56' N, 3º 33' W; 580 m a.s.l.) during May-June 2013. The study area is dominated by orange groves, in which blackbirds usually nest. For a detailed description of the population, see [34].

The Common Blackbird is a medium-sized passerine frequently used as a model species in egg-recognition experiments, e.g. [18, 35–39], which is a potential host of the Common Cuckoo but is not actually parasitized. However, blackbirds are able to recognize and eject alien eggs artificially introduced in their nests [18, 36, 40, this study]. We actively searched for blackbird nests in the study area throughout the breeding season from early May to the end of June. Once a nest was located, we checked it to determine its content and status, and visited it every two days to record laying date and clutch size.

### Models eggs

Non-mimetic model eggs used in our study were similar in size and colour but not in weight. We used natural eggs (from naturally deserted nests) whose weight was modified by adding a mixture of sand and silicone. Collected eggs were emptied through a small hole (< 3 mm) made in the eggshell using a needle and then filled with the sand-silicone mixture. We varied the sand-silicone ratio in the egg to achieve different weights in completely filled eggs. Finally, the small hole was sealed with silicone. Three different treatments were created in terms of egg mass: *(i)* light eggs (mean ± SE; 3.0 ± 0.1 g; N = 14): on average 55% lighter than natural blackbird eggs, similar to egg mass of Common Cuckoos that parasitize Rufous-tailed scrub robins, *Cercotrichas galactotes* [41]; *(ii)* normal-weight eggs: the same weight as the blackbird eggs (mean ± SE; 6.7 ± 0.2 g; N = 14.); and *(iii)* heavy eggs: on average 49% heavier than natural blackbirds eggs (mean ± SE; 10.0 ± 0.3 g; N = 16). This increase in egg mass was not disproportionately large in comparison with that of our population (mean ± SE; 6.95 ± 0.05 g; range min = 5.5 g, max = 7.8 g; N = 154) or other populations of this species (7.58 ± 0.03 g; N = 772; range not provided; [42]). In fact, similar differences are found in egg mass for some host-parasite systems in which the parasitic egg can be 48.5% heavier than that of host egg [43]. We created an additional group of nests (hereafter natural group) for which we followed the same procedure in relation to visits, checking, and filming but which were not experimentally parasitized.

Model eggs were coloured red with acrylic paint the day before being placed in the experimental nest. We used non-mimetic eggs to ensure that model eggs were easily recognized and to standardize the colour of all manipulated eggs. Model eggs painted red have been frequently used in egg-recognition experiments in which it has been demonstrated that they are good non-mimetic model eggs [8, 18, 39, 44, 45]. Each model egg was used only in one trial and discarded afterwards.

### Experimental design

**Fig 1:**
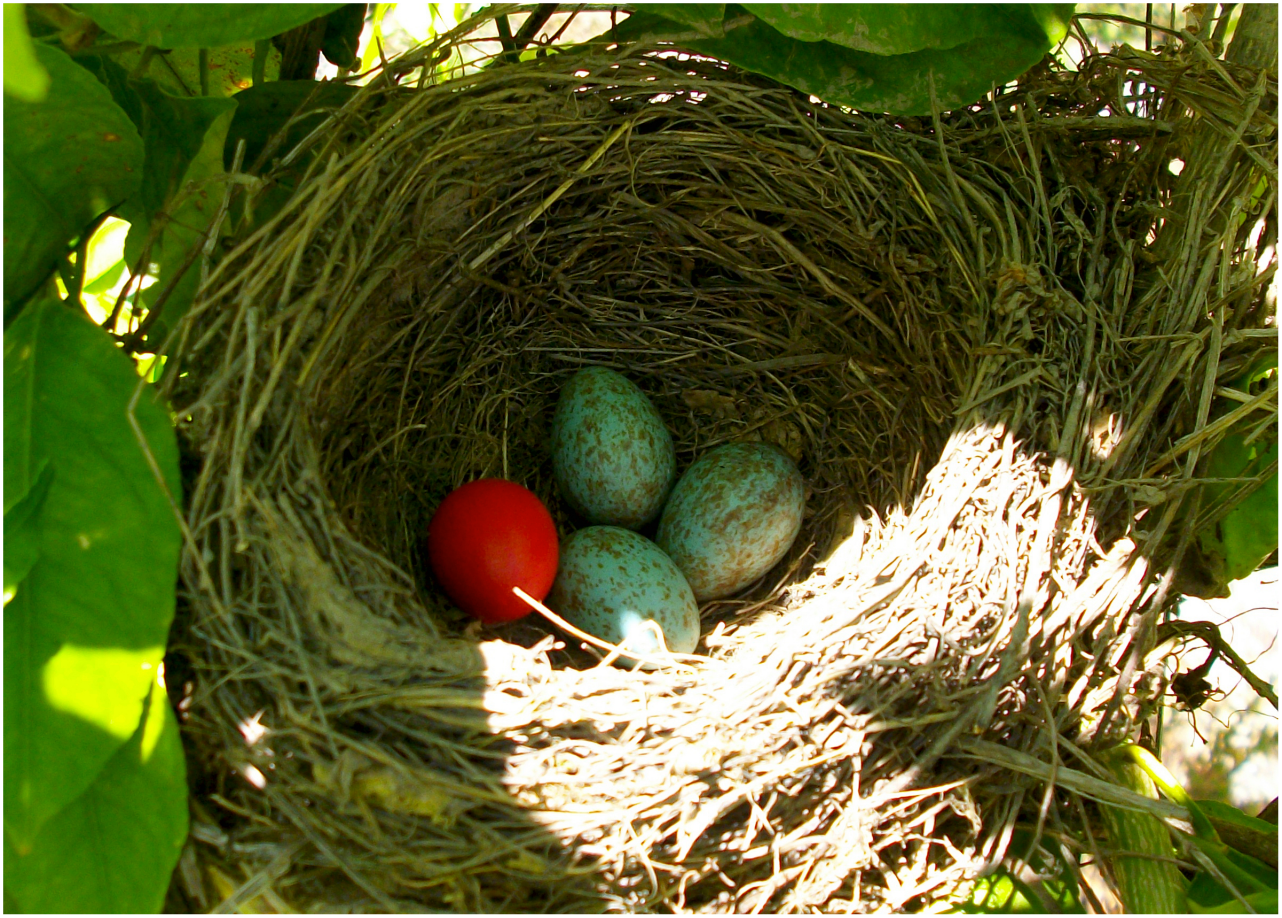
Photo of a blackbird nest containing three natural eggs parasitized with a normal-weight non-mimetic egg.

Blackbird nests were experimentally parasitized by using non-mimetic model eggs of different weights (see above). Model eggs were introduced into the nests during the laying (minimum of two eggs laid) or incubation stages (Fig 1). Previous studies have shown that blackbirds reject experimental eggs at similar rates in both the laying and the incubation stages, e.g. [21, 35, 36, 46]. Each nest was assigned randomly to one of the three egg mass treatments (light, normal-weight or heavy eggs; see above).

A video camera (Panasonic HDC-SD40) was placed near the nest (1.5–2.5 m) just after the introduction of the model egg in order to film the response of blackbirds to the model egg for the following two hours. We used a standardized procedure previously used in other studies of the incubation behaviour of this species e.g. [18, 47]. For this species, the placement of a camera near the nest does not affect their behaviour in relation to egg-introduction experiments [18].

We checked for the introduced egg in the nest after the two recording hours. If the model egg was in the nest, we checked it again after 24 h and continued this procedure for the following five days. This (and the analysis of recordings) enabled us to determine the ejection time of each experimental egg. When the model egg disappeared, we assigned the ejection time considering that the ejection occurred between the last two visits adding 12 hours to the time (in hours) of the last visit in which the introduced model egg was still present. If the egg remained intact in the nest for five days after its introduction, we considered it to have been accepted. In each visit, we inspected all eggs (host and experimental), looking for ejection costs (cracks or broken eggs) and recognition costs (one or more blackbird eggs mistakenly ejected). We used five days for our trials following the procedure used in other egg-rejection experiments conducted in thrushes, e.g. [18, 46, 48, 35–37].

The recordings were visualized using the KM Player 3.5 Plus software. We compiled information on egg-recognition behaviour by analysing different variables of recordings: (1) first contact touches (number of times the female touched the eggs from their arrival to the nest until she sat on the nest for the first visit), (2) first contact touches per visit (similar to the previous variable but for the complete filmed period -2 hours-, corrected by the number of visits) and (3) incubation touches (number of times the female touched the eggs while incubating per hour). Female touches have been used in several studies of parasitism as a measure of recognition of parasitic egg, e.g. [13, 18, 29]. In this study, we used differences in female touches between non-mimetic and natural eggs as an indicator of recognition.

### Statistical procedures

We differentiated between two different ejection variables: (*i*) immediate ejection: female response against the parasitic egg within the two hours after parasitism and (*ii*) long-term ejection: female response to the parasitic eggs considering the whole experimental period (five days). To assess the effect of our treatment for each of these two types of ejection variables, we used generalized linear models (binomial error and logit link function). Recognition variables were analysed using generalized linear models (poisson error and log link function). Differences between levels were compared using the “multcomp” R package for generalized linear models. All analyses were performed using R version 3.1.1.

### Ethical Note

The filming of adults did not negatively affect blackbird egg hatchability relative to natural nests and none of the nests used in this study was deserted. This research was conducted according to national (Real Decreto 1201/2005, de 10 de Octubre) and regional (permissions provided yearly by Consejería de Medio Ambiente de la Junta de Andalucía) guidelines.

## Results

We performed our experiment in 58 blackbird nests. Four of them could not be used in our long-term ejection analyses because they were predated before the end of the trial (the fifth day after the egg introduction).

Regarding egg recognition, we found no significant differences among the tree non-mimetic model eggs for first contact touches (χ^2^ = 0.91, df = 2, p = 0.6, N = 44; Table 1) or first contact touches per visit (χ^2^ = 0.06, df = 2, *p* = 1.0, N = 44; Table 1). Furthermore, the weight of the model egg did not significantly influence the number of touches during the incubation (χ^2^ = 3.03, df = 2, *p* = 0.2, N = 44; Table 1). These results allow us to assume that the three types of model eggs were equally recognized regardless of weight. Model eggs (heavy, light, and normal-weight eggs) taken together were significantly more pecked by females than their own blackbird eggs (natural eggs) for the first contact after being away from the nest both during the first visit (z = -4.485, *p <* 0.001, N = 58; Table 1) and considering all visits together (number of touches per visit, z = -4.055, *p <* 0.001, N = 58; Table 1). We also found differences for female touches during incubation between model and natural eggs (z = 12.38, *p* < 0.001, N = 58; Table 1) indicating that natural eggs were more touched during the incubation than model eggs.

**Table 1:** Mean ± SE of female recognition touches for the first visit, touches per visit, and during incubation in the three non-mimetic model eggs (light, normal-weight, and heavy eggs) and natural blackbirds eggs.

Treatment	N	First contact touches	First contact touches per visit	Incubation touches
Light	14	2.57 ± 0.58	2.71 ± 0.45	33.00 ± 10.18
Normal-weight	14	3.14 ± 0.98	2.57 ± 0.82	33.01 ± 7.66
Heavy	16	2.69 ± 0.63	2.69 ± 0.67	29.99 ± 5.01
Natural	14	0.43 ± 0.29	0.57 ± 0.23	55.48 ± 23.66

Considering the immediate ejection rate, we found a significant effect of our treatment in the immediate ejection rate (χ^2^ = 9.58, df = 2p = 0.01, N = 44). None of the heavy eggs was ejected during the filming period (within the first two hours). In contrast, 36.5% of light eggs and 28.6% of normal-weight eggs were ejected during this period. These data seem to indicate that heavy eggs are harder to eject from the outset.

**Fig 2:**
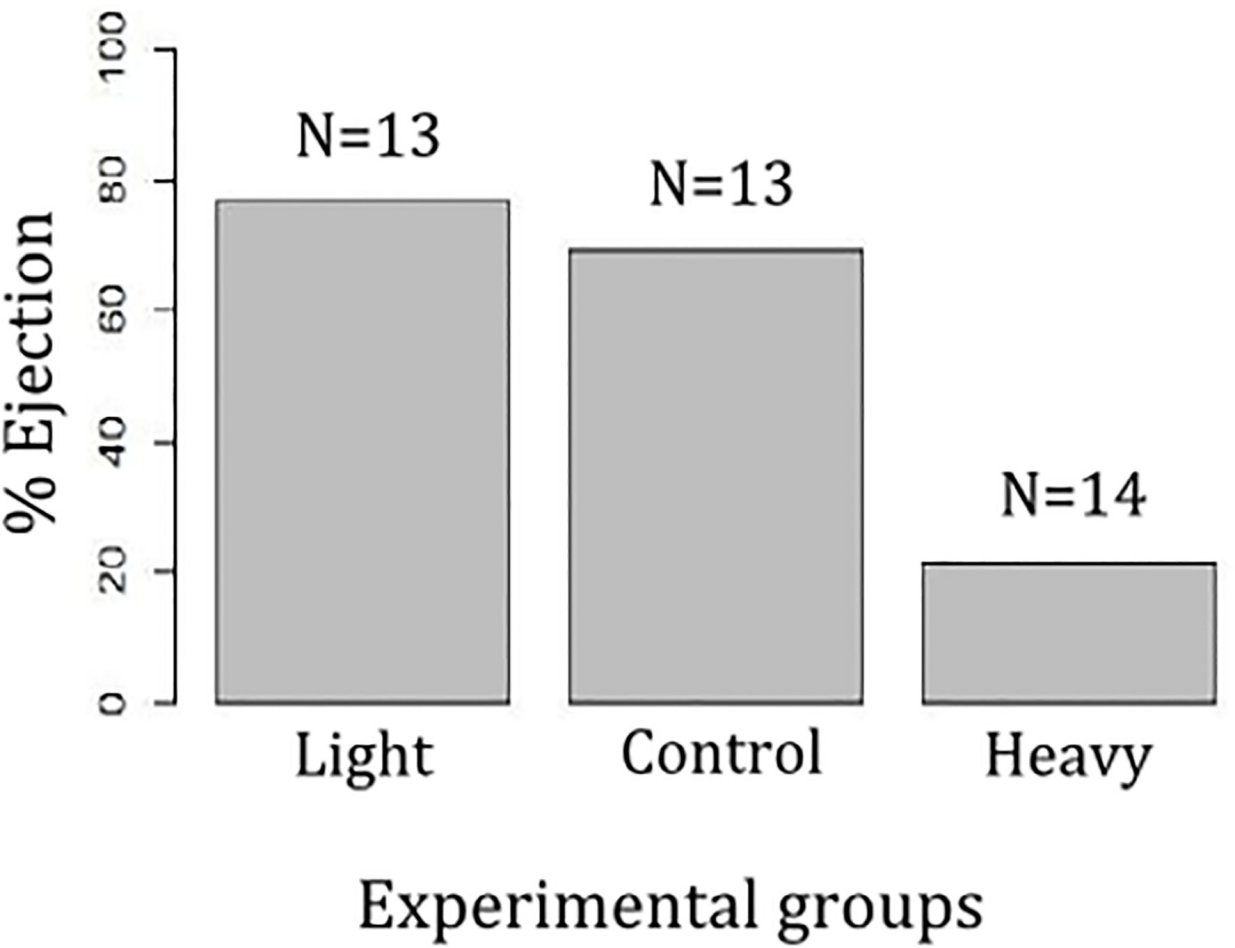
Ejection rate for each experimental treatment: light, normal-weight, and heavy eggs. Sample sizes for each treatment are shown at the top of each column.

We found significant differences among our three treatments of parasitized nests for long-term ejection rate (χ^2^ = 10.41, df = 2, *p* = 0.01, N = 40; Fig 2). As we predicted, the main effect of our treatment in the ejection rate was due to heavy eggs. Thus, the ejection rate was significantly lower when blackbird nests were parasitized with heavy eggs than in those cases containing normal-weight (Tukey HSD: *p* = 0.046) or light ones (Tukey HSD: *p* = 0.02). No differences in long-term ejection rate were found between normal-weight nests and those parasitized with light eggs (Tukey HSD: *p* = 0.9).

Filmed ejection events (N = 9) showed that blackbirds ejected the experimental egg by grasping it with their bills in all cases. None of the eggs (experimental or natural) presented cracks and none of the host eggs were mistakenly ejected, indicating that there were no recognition or ejection costs.

## Discussion

Our results show that experimental eggs introduced into the blackbird nests were recognized by the blackbirds as foreign eggs and this recognition was not affected by the egg mass (Table 1). Despite this, heavy eggs were more frequently accepted than the other two experimental egg types (Fig 2), indicating that egg mass critically affects egg rejection. This work supports previous studies highlighting that hosts may recognize more eggs than they finally reject [13, 24, 26–28, 29, 49].

Egg rejection is a complex process in which three phases can be considered: recognition, decision whether to reject or not, and action, (see Fig 6 in [13]). If egg recognition does not occur the outcome of parasitism is acceptance of the parasitic egg. Red model eggs used in our study seem to be easily recognized, as indicated by the fact that blackbirds pecked non-mimetic model eggs more frequently than their own (Table 1) in accordance with previous results [18]. Incubation touches differed between models and natural eggs, thus model eggs were pecked less frequently during incubation than natural eggs. Likely, this variable is not a good proxy to quantify egg recognition because incubation touches are related to egg turning and, thus, inherently related to the incubation process [50, 51, 52]. According to our results, model eggs were recognized from the first visit to the nest and they were less turned than natural eggs during incubation. Furthermore, first contact touches for the first visit and per visit of the experimental red egg did not differ among all the three experimental groups (light, normal-weight, and heavy), suggesting that experimental eggs were equally recognized, regardless of their weight. As expected, these results confirm that other cues (mainly visual cues such as colour patterns) rather than egg mass are critical for hosts to differentiate parasitic eggs, especially in open nests [3, 7, 14, 16, 17]. However, although model eggs were recognized at similar rates, light and normal-weight eggs were ejected at higher rates than were heavy eggs (Fig 2), this constituting the first experimental demonstration that a trait which does not affect recognition (eggs mass) may lead to the decision of acceptance instead of ejection.

Several studies have shown that egg discrimination is not always followed by egg rejection (see references above). Antonov et al. [29] suggested that the acceptance of previously pecked eggs in eastern olivaceuos warblers *Hippolais pallida* was due to the impossibility of puncturing the parasitic egg by this small-sized host. However, other studies have suggested that egg rejection has to be considered a conditional process in which, once the alien egg has been recognized, the individual host response against the parasitic egg may be modulated by the perceived risk of parasitism and potential rejection costs [13, 25, 44, 53, 54]. Soler et al. [13] demonstrated that the frequent cases of discrimination without ejection of experimental eggs found in the rufous-tailed scrub robin, a common host of the common cuckoo in southern Spain, were not due to the impossibility of ejecting the experimental egg. That study showed that when scrub robins were parasitized with soft eggs (real house sparrow eggs), 80% of the pecked eggs were not ejected, either. Thus, discrimination without ejection was not the consequence of the physical inability of birds to puncture-eject them, but the consequence of a decision against ejecting due to low motivation.

Clearly, in the case of blackbirds confronted to heavy model eggs, discriminated eggs that were not ejected was not because of the impossibility of ejection because the eggs were of the same size as the ones of the other two experimental groups and so the mechanical difficulty of grasping them was the same. Although a greater egg mass makes it difficult to handle the experimental egg, blackbirds are capable of ejecting heavy eggs (Fig 2). Furthermore, recordings did not show failed ejection attempts (i.e. eggs that fall from the female bill while trying to eject them). Instead, blackbirds immediately recognize the introduced model egg and they pecked it softly before starting the incubation. Just as previously suggested by [13], higher motivation to eject is required for the host to take on higher costs and continue the rejection process. In the scrub robin, soft pecking was suggested to constitute tentative testing behaviour and that it was not part of the recognition process because only the experimental egg was pecked, indicating recognition. Presumably, touching the eggs by blackbirds has the same function once the egg is recognized.

Blackbirds had no problem ejecting either normal-weight or light eggs by grasping them with their bills. In fact, 36.5% of light and 28.6% of normal-weight eggs were ejected during the first two hours after parasitism but no heavy egg was ejected during this period. These trends were confirmed regarding the long-term ejection rates as only 21.4% of heavy eggs were ejected. One explanation for these results could be that a greater mass limits the handling of the egg and makes it more difficult (but still possible) to be removed from the nest. Accordingly, hard work would be needed to eject a heavy egg from the nest and this may imply a higher risk of breaking one or more of their own eggs, so more motivation would be required to assume these higher costs of ejecting a heavier egg. In our study, none of the blackbird eggs presented cracks or disappeared from the nest. As shown in the recordings, this may be due to females slightly checking the parasitic egg with their bill before attempting to eject it. These soft checks were in no case failed attempts to expel the egg, which indicates that acceptance of a heavy egg is due to a decision not to assume the potential cost of ejecting a heavy egg. This may indicate that females that decide to eject the heavy egg are those in good physical condition and thus capable of ejecting the egg without breaking any of their own eggs. The females that decide not to eject the heavy egg would be the ones that, after testing (i.e. touching and moving) the egg, regard the risk of destroying one or more of their own eggs to be high and therefore decide against rejection.

In conclusion, blackbirds accepted some previously recognized heavy eggs, probably because their motivation was not high enough to assume the possible higher ejection costs of ejecting a heavy egg. These results highlight the importance of distinguishing recognition and rejection in brood-parasitism studies and considering the conditional component of the host response.

## Supporting Information

S1 Table Summary of female responses to experimental parasitism (S1A Table) and number of touches used to determine recognition of experimental eggs (S1B Table). (PDF)
