# Supplementary material for: Could a Factor That Does Not Affect Egg Recognition Influence the Decision of Rejection?
Source: PLoS One. 2015 Aug 21;10(8):e0135624. doi: 10.1371/journal.pone.0135624 (PMC4546668; doi:10.1371/journal.pone.0135624)
Supplement: S1 Table — (PDF) [file pone.0135624.s001.pdf]

Could a factor that does not affect egg recognition influence the decision of rejection?

Francisco Ruiz-Raya, Manuel Soler, Lucía Ll. Sánchez-Pérez and Juan Diego Ibáñez-Álamo

Supporting Information 1 Summary of female responses to experimental parasitism and number of touches used to determine recognition of experimental eggs.

S1A Table Responses of female blackbirds (immediate ejection and long-term ejection) to the experimental model eggs: light (LIG), normal-weight (NOR) and heavy (HEA).

Nest ID	Treatment	Immediate ejection	Long-term ejection
185	NOR	no	no
190	HEA	no	no
197	LIG	yes	yes
202	LIG	no	yes
210	LIG	no	no
216	NOR	no	no
220	LIG	yes	yes
222	HEA	no	no
225	HEA	no	no
227	LIG	yes	yes
228	HEA	no	no
229	HEA	no	no
230	LIG	no	no
231	LIG	no	no
232	NOR	no	yes
233	HEA	no	no
239	NOR	no	yes
242	NOR	no	yes
243	HEA	no	no
245	LIG	yes	yes
246	HEA	no	no
248	NOR	no	no

Nest ID	Treatment	Immediate ejection	Long-term ejection
250	NOR	no	no
254	LIG	no	-
257	HEA	no	no
260	HEA	no	no
262	HEA	no	yes
267	NOR	yes	yes
268	NOR	yes	yes
269	LIG	no	yes
270	LIG	no	yes
272	NOR	no	yes
277	HEA	no	yes
282	NOR	no	yes
283	LIG	yes	yes
285	HEA	no	yes
294	NOR	no	-
297	LIG	no	yes
299	NOR	yes	yes
301	LIG	no	yes
302	HEA	no	no
303	HEA	no	-
305	NOR	yes	yes
310	HEA	no	-

S1B Table Data used to analyse recognition of model (MODEL) and natural eggs (NAT). Touches per visit, first contact touches and incubation touches for the four treatments: light (LIG), normal-weight (NOR), heavy (HEA) and natural (NAT) eggs.

Nest ID	Egg	Treatment	Touches per visit	First contact touches	Incubation touches
185	MODEL	NOR	9	14	83,75
190	MODEL	HEA	0	0	8,75
197	MODEL	LIG	4	7	89,33
202	MODEL	LIG	2	2	42,76
210	MODEL	LIG	4	4	0,00
216	MODEL	NOR	2	0	21,59
220	MODEL	LIG	5	3	16,72
222	MODEL	HEA	1	2	17,76
225	MODEL	HEA	0	1	34,87
227	MODEL	LIG	2	0	20,62
228	MODEL	HEA	2	3	20,81
229	MODEL	HEA	0	0	0,00
230	MODEL	LIG	1	1	0,00
231	MODEL	LIG	2	0	0,00
232	MODEL	NOR	1	0	44,17
233	MODEL	HEA	1	0	62,33
235	NAT	NAT	0	1	0,00
238	NAT	NAT	1	4	59,83
239	MODEL	NOR	10	6	88,88
240	NAT	NAT	0	0	303,26
241	NAT	NAT	1	0	8,88
242	MODEL	NOR	1	3	28,96
243	MODEL	HEA	5	10	10,88
245	MODEL	LIG	3	4	33,90
246	MODEL	HEA	1	3	23,24
248	MODEL	NOR	1	2	11,82
250	MODEL	NOR	0	0	0,00
254	MODEL	LIG	1	4	132,10
257	MODEL	HEA	0	1	30,19

Nest ID	Egg	Treatment	Touches per visit	First contact touches	Incubation touches
260	MODEL	HEA	0	4	0,00
262	MODEL	HEA	8	4	52,19
266	NAT	NAT	0	0	29,40
267	MODEL	NOR	0	3	67,95
268	MODEL	NOR	3	3	20,41
269	MODEL	LIG	4	0	17,54
270	MODEL	LIG	0	0	17,08
272	MODEL	NOR	2	1	25,20
277	MODEL	HEA	6	3	37,69
280	NAT	NAT	0	0	37,54
281	NAT	NAT	0	0	11,63
282	MODEL	NOR	0	5	13,37
283	MODEL	LIG	6	3	29,75
285	MODEL	HEA	7	2	54,98
291	NAT	NAT	0	0	23,65
293	NAT	NAT	0	0	0,00
294	MODEL	NOR	0	4	8,60
297	MODEL	LIG	1	5	14,25
299	MODEL	NOR	2	3	40,68
301	MODEL	LIG	2	3	18,53
302	MODEL	HEA	3	4	41,06
303	MODEL	HEA	1	1	27,49
305	MODEL	NOR	0	0	6,74
309	NAT	NAT	1	1	210,04
310	MODEL	HEA	4	5	57,55
316	NAT	NAT	3	0	19,01
317	NAT	NAT	1	0	33,11
319	NAT	NAT	0	0	16,74
321	NAT	NAT	0	0	23,68
